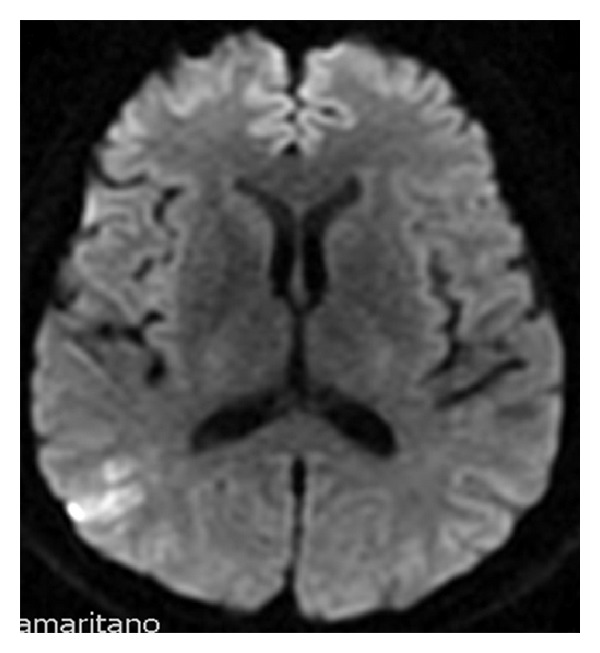# Native Valve Endocarditis due to *Enterococcus hirae* Presenting as a Neurological Deficit

**DOI:** 10.1155/2013/636070

**Published:** 2013-09-16

**Authors:** Renato Anghinah, Rafael Gustavo Sato Watanabe, Mateus Mistieri Simabukuro, Carla Guariglia, Lécio Figueira Pinto, Daniella Costa de Menezes e Gonçalves

**Affiliations:** ^1^Neurology Department at Hospital Samaritano, São Paulo, SP, Brazil; ^2^Neurology Department at Clinic Hospital of Medicine School, University of São Paulo (HCFMUSP), São Paulo, SP, Brazil; ^3^University of São Paulo Medical School, Division of Neurology of the Medicine School, Rua Itacolomi 333/83, São Paulo, SP, Brazil; ^4^Infectology Department at Hospital Samaritano, São Paulo, SP, Brazil

## Abstract

*Enterococcus hirae* is a rare isolate in clinical specimens. We describe a case of native aortic valve endocarditis in a 56-year-old man. This is the third reported case of endocarditis due to this organism, the first without recurrence of endocarditis and the first presenting as a neurological deficit.

## 1. Introduction 

Enterococci are Gram-positive bacteria that are established as major nosocomial pathogens and really important due to the development and transmission of antibiotic resistance traits [1]. The enterococci are the third most common cause of infective endocarditis, accounting for 5 to 15% of cases, and associated with a mortality of 20 to 30% [1]. The majority of enterococcal strains isolated from human specimens belong to the *Enterococcus faecalis *(80%) and *Enterococcus faecium *(10%) species. *E. hirae *accounts for less than 1% of enterococcal species in human clinical samples [2]. This species is known to cause infections in a range of young farmed species and psittacine birds [3, 4], but it is very rare in humans. The first description of *E. hirae *infection in humans, by Gilad et al. in 1998, reported a case of septicemia in a patient with end-stage renal disease undergoing hemodialysis [5]. We describe a case of native aortic valve endocarditis in a 56-year-old man. This is the first reported case of endocarditis presenting as a neurological deficit.

## 2. Case Presentation

**Figure 1:**
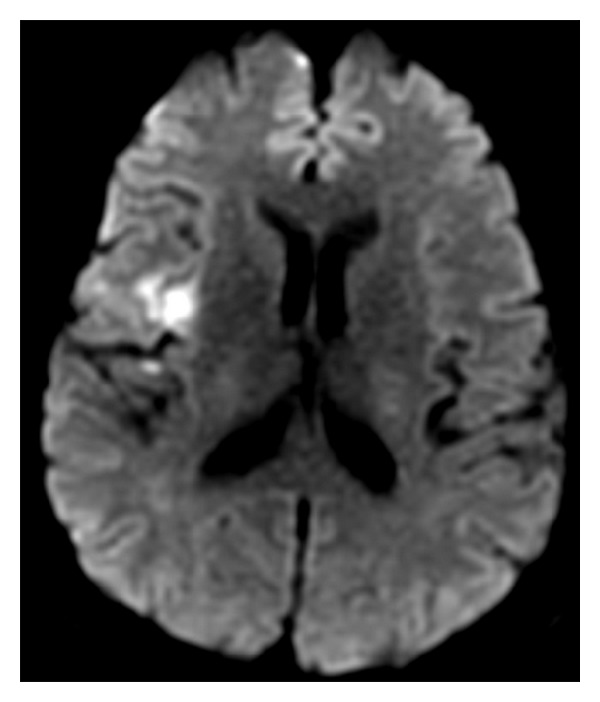


In August 2011, a 56-year-old man presented with a symptom of slurred speech from the moment he woke up. He had a previous medical history of hypertension, diabetes, hypercholesterolemia, cardiac arrhythmia with surgical ablation, and surgical removal of a gastric leiomyoma. In the previous month he lost 8 Kg and felt generalized fatigue, depressive symptoms, and evening fever (but not measured at home). On examination, our patient was dysarthric and dysphagic, associated with a complete left hemiparesis (grade IV). His temperature was 38°C, and pulse rate was 80 beats per min; blood pressure was 110/70 mmHg and cardiac examination was normal, without cardiac murmurs. The initial brain MRI (Figure 1) showed hyperintensity in T2/FLAIR and diffusion-weighted images at the right insula, compatible with ischemic lesion. Transesophageal echocardiogram revealed patent foramen ovale and the presence of an irregular vegetative image between the commissures of the aortic valve at its maximal extension with 34 mm. The patient was initially treated with oxacillin (150 mg/Kg/day) plus gentamicin (3 mg/Kg/day). Cerebrospinal fluid revealed 35 cells, 75% neutrophils, protein 52, glucose 62, and lactate 23; then ampicillin was associated (150 mg/Kg/day) considering the diagnosis of listeria. After 10 days of investigation and treatment, blood culture was positive for *Enterococcus hirae*. Therefore the antibiotic regimen was altered to ampicillin plus rifampin (7.5 mg/Kg/day).

In a control brain MRI in the diffusion-weighted image a new lesion was found in the right parietal cortex (Figure 2); a new transesophageal echocardiogram showed aortic valve reflux of moderate to important intensity, vegetative image at its maximal extension with 23 mm, and mitral valve with moderate reflux associated with an image compatible with a vegetation of 16 to 21 mm. He promptly underwent a successful cardiac surgery for the replacement of the aortic valve by a biological valve, plastic of the mitral valve, and correction of the foramen ovale. The patient improved his clinical condition and laboratory markers, with minimal alteration on neurological exam; he completed a total of 4 weeks of intravenous antibiotic (ampicillin plus rifampicin) and was discharged with amoxicillin plus rifampin for another 2 weeks. At last followup in February, 2012, he had resumed work and was asymptomatic.

## 3. Discussion

*Enterococcus* is Gram-positive bacteria. They belong to the microbiota of animals and humans as commensal colonization. Nonetheless, both *E. faecalis* and *E. faecium* are frequently associated with human infection such as bacteremia, endocarditis, and urinary tract infections. *E. hirae *is a pathogen frequently associated with infections in animal species such as birds. In humans the enterococci are usually commensals of the digestive tract causing infections in less than 1% [5–7]. The first report of human infection caused by *E. hirae *was described by Gilad et al. [5] in 1998 in a case of septicemia in a patient with end-stage renal disease under hemodialysis. Regarding endocarditis caused by *E. hirae *our patient is the third case described so far. We believe that the importance of this case is due to the fact that it was the first treated without recurrence. 

Poyart et al. [8] in 2002 described a case of a patient with infectious endocarditis in a native aortic valve by *E. hirae *treated initially with the association of ampicillin (200 mg/Kg/day) plus gentamicin (3 mg/Kg/day) for 4 weeks, and 2 weeks after the initiation of antibiotics, rifampin (25 mg/Kg/day) was associated for 2 weeks. The patient was released from the hospital in use of oral ampicillin and rifampin for another 3 weeks. These antibiotics were ineffective in sterilizing the aortic vegetation and 3 months after suspension of antibiotics there was a relapse of the infectious endocarditis. A different antibiotic program was initiated: vancomicin 60 mg/Kg/day plus gentamicin 3 mg/Kg/day for 6 weeks; it was associated with valve exchange after 10 days of antibiotics and followed by oral amoxicillin 6 g/day for a total of 8 weeks. A similar outcome was reported by Talarmin et al. [9] in 2011; first amoxicillin 200 mg/Kg/day (IV) plus gentamicin 3 mg/Kg/day (IV) for 2 weeks was introduced followed by amoxicillin 200 mg/Kg/day (IV) plus rifampin 20 mg/Kg/day for a total of 6 weeks. After 4 months of the treatment ended, the endocarditis in the prosthetic valve relapsed; it was then restarted amoxicillin 200 mg/Kg/day IV for 6 weeks plus gentamicin 3 mg/Kg/day (IV) for 2 weeks associated with rifampin for 4 weeks. Rifampin seems to be indicated in the treatment of *Staphylococcus aureus *in prosthetic valve, probably by its ability to penetrate the biofilm of the bacteria [10] although this was not extensively studied for the *Enterococcus *species. The ideal therapeutic for *E. hirae *infections remains unknown, but as we demonstrated, the hypothesis that rifampin is beneficial to the treatment regimens seems plausible. According to previous data the infectious endocarditis by *E. hirae *seemed to cause severe endocarditis [8, 9] with bad prognosis (valve exchange, recurrence); because our patient needed valve exchange by neurological cause (new lesions in MRI, despite the lack of symptoms), we cannot be sure that it would not relapse without the valve exchange. Therefore the procedure of valve exchange should be considered in endocarditis caused by this pathogen. 

## Figures and Tables

**Figure 2:**